# Activity of Telavancin against Staphylococcus aureus Isolates, Including Those with Decreased Susceptibility to Ceftaroline, from Cystic Fibrosis Patients

**DOI:** 10.1128/AAC.00956-18

**Published:** 2018-08-27

**Authors:** Melanie Roch, Maria Celeste Varela, Agustina Taglialegna, Warren E. Rose, Adriana E. Rosato

**Affiliations:** a Department of Pathology and Genomic Medicine, Center for Molecular and Translational Human Infectious Diseases Research, Houston Methodist Research Institute, Houston, Texas, USA; b School of Pharmacy, University of Wisconsin—Madison, Madison, Wisconsin, USA

**Keywords:** cystic fibrosis, chronic infections, MRSA, telavancin

## Abstract

Methicillin-resistant Staphylococcus aureus (MRSA) acquisition in cystic fibrosis (CF) patients confers a clinical outcome worse than that in non-CF patients with an increased rate of declined lung function. Telavancin, an approved lipoglycopeptide used to treat infections due to S. aureus, has a dual mode of action causing inhibition of peptidoglycan synthesis and membrane depolarization. MRSA infections in CF patients remain an important problem with no foreseeable decline in prevalence rates. Although telavancin is currently in clinical use for the treatment of complicated skin infections and hospital-acquired pneumonia, the activity against S. aureus infections in CF patients has not been investigated. In this work, we studied the activity of telavancin against CF patient-derived S. aureus strains collected from geographically diverse CF centers in the United States. We found that the telavancin MIC_90_ was 0.06 μg/ml, 8-fold lower than the ceftaroline or daptomycin MIC_90_ and 25-fold lower than the linezolid and vancomycin MIC_90_. We demonstrate that telavancin at serum free concentrations has rapid bactericidal activity, with a decrease of more than 3 log_10_ CFU/ml being achieved during the first 4 to 6 h of treatment, performing better in this assay than vancomycin and ceftaroline, including against S. aureus strains resistant to ceftaroline. Telavancin resistance was infrequent (0.3%), although we found that it can occur *in vitro* in both CF- and non-CF patient-derived S. aureus strains by progressive passages with subinhibitory concentrations. Genetic analysis of telavancin-resistant *in vitro* mutants showed gene polymorphisms in cell wall and virulence genes and increased survival in a Galleria mellonella infection model. Thus, we conclude that telavancin represents a promising therapeutic option for infections in CF patients with potent *in vitro* activity and a low resistance development potential.

## Abstract (precis)

Methicillin-resistant Staphylococcus aureus (MRSA) acquisition in cystic fibrosis (CF) patients confers a clinical outcome worse than that in non-CF patients with an increased rate of declined lung function. Telavancin, an approved lipoglycopeptide used to treat infections due to S. aureus, has a dual mode of action causing inhibition of peptidoglycan synthesis and membrane depolarization.

## INTRODUCTION

Methicillin-resistant Staphylococcus aureus (MRSA) is an important infectious human pathogen responsible for diseases ranging from skin and soft tissue infections to life-threatening endocarditis in both hospital and community settings. β-Lactam resistance in MRSA involves the acquisition of penicillin-binding protein 2a (PBP 2a), which has a low affinity for β-lactams and can mediate cell wall assembly when the normal staphylococcal penicillin-binding proteins (PBPs 1 to 4) are inactivated by these agents (1). S. aureus is one of the earliest and more prevalent pathogens, colonizing the respiratory tract and then causing infection in people with cystic fibrosis (CF). This severe, autosomal recessive disease affects several organs, notably, the lungs, predisposing these patients to reduced respiratory function and infections with potentially severe consequences. According to recent data from the CF Foundation Patient Registry, the prevalence of methicillin-susceptible S. aureus (MSSA) in the United States is about 70%, while that of MRSA is 26%. These values for MRSA compare to values of 13% in Europe, 6% in Canada, and 3% in Australia (2, 3). Emerging research has demonstrated that MRSA infections have a significant clinical impact on individuals with underlying chronic diseases, such as CF, where antibiotic pressure and metabolic adaptations may favor the ability of S. aureus to establish long-term persistence and resistance (4). Additional mechanisms reported in CF lung and other chronic MRSA infections reduce antibiotic activity, including small-colony-variant (SCV) adaptation, biofilm formation, and growth under anaerobic conditions, which are associated with higher rates of antimicrobial treatment failure (5–8). Moreover, in patients with CF, chronic pulmonary infections with MRSA and their exacerbations were shown to be associated with a decline in lung function and a clinical outcome worse than that in non-CF patients (9). In this context, additional data regarding the antibiotic susceptibility of strains from CF patients are urgently needed to enhance treatment options against multidrug-resistant strains and to try to eradicate MRSA from their lungs.

Telavancin (TLV) is a lipoglycopeptide antibiotic approved by the FDA in 2009 for the treatment of complicated skin and skin structure infections and in 2013 for the treatment of cases of nosocomial pneumonia (hospital-acquired pneumonia [HAP]/ventilator-associated pneumonia [VAP]) (10) suspected to be caused by MRSA. TLV was developed from the parent molecule vancomycin (11), and its bactericidal action involves membrane depolarization and the inhibition of peptidoglycan (PG) synthesis (12, 13) at late-stage PG precursors, including lipid II; however, the precise mode of action of TLV on the membranes of Gram-positive bacteria has not yet been determined (6–8).

*In vitro* studies have demonstrated that TLV has activity against MRSA, including vancomycin-intermediate S. aureus (VISA) strains (10, 14). However, clinical data on TLV activity against MRSA strains isolated from patients with chronic diseases, such as cystic fibrosis, are not yet available. These strains are well-known to have an altered metabolism and possess multidrug resistance due to their chronic habitation of the CF patient lung and prolonged, repeated exposures to antibiotic treatments in the CF lung environment (15).

We hypothesized that TLV may represent a valid option for the treatment of CF patient-derived MRSA and MSSA infections. Therefore, the purpose of this study was to characterize by *in vitro* and *in vivo* approaches the antimicrobial activity of TLV against S. aureus chronic infection strains, in particular, MRSA strains, isolated from patients at diverse CF centers in the United States. Lastly, we aimed to understand TLV resistance selection within an S. aureus population derived from a CF patient background.

## RESULTS

### Susceptibility of cystic fibrosis patient-derived MRSA/MSSA strains to telavancin.

The CF patient-derived S. aureus strains used in the present study were of either the wild-type or small-colony-variant (SCV) phenotype, identified at the time of culture, and were obtained from three different CF centers. We screened a total of 333 strains; those collected at the Houston Methodist Research Institute, 103 in total, were distributed as 37 MRSA and 66 MSSA strains, while strains originating from UW Health comprised 72 MRSA and 10 MSSA strains. The ones obtained from the Center for Global Infectious Disease Research at Seattle, WA, comprised 148 total S. aureus strains, with 44 being MRSA strains and 104 being MSSA strains. As shown in Table 1, TLV displayed activity against all 333 strains derived from CF patients at three different CF centers from across the United States; the majority of these S. aureus strains were isolated from adult patients, with only 41 strains being found in children. Furthermore, we tested the activity of TLV against 23 MRSA strains; 20 displayed intermediate resistance to ceftaroline (CPT^ir^; MICs, 1.5 to 2 μg/ml) and 3 had high-level resistance to ceftaroline (CPT^hr^; MIC, 32 μg/ml).

**TABLE 1:** Telavancin MIC_90_ of CPT for 333 S. aureus strains from CF patients^*a*^

Strain	MIC (μg/ml)^*b*^				
	CPT	TLV	DAP	VAN	LZD
MSSA (*n* = 180)	0.5 (100)	0.06 (100)	0.25 (100)	1.0 (100)	1–2 (99.4)
MRSA (*n* = 153)					
CPT^s^ (*n* = 130)	<1.5 (85)				
CPT^ir^ (*n* = 20)	1.5–2 (13)	0.06 (100)	0.5–1 (100)	1–1.5 (100)	1–2 (98.1)
CPT^hr^ (*n* = 3)	>32 (2)				

a The MIC_90_ of CPT was determined by the microdilution method in Mueller-Hinton broth supplemented with polysorbate 80 (0.002%) and was compared to the MIC_90_s of the DAP, VAN, LZD, and CPT agents following CLSI guidelines.

b Values in parentheses represent the percentage of isolates with the indicated MIC.

CPT is a new β-lactam antibiotic that specifically targets PBP 2a in MRSA. Although a high level of resistance to CPT remains rare, intermediate resistance is more prevalent in patients with chronic infections. Among all strains, the TLV MIC_90_ was 0.06 mg/liter, i.e., 8-fold lower than the daptomycin (DAP) and CPT MIC_90_ and 25-fold lower than the linezolid (LZD) and vancomycin (VAN) MIC_90_. In the strains with reduced CPT susceptibility, the TLV MIC_90_ was 0.06 μg/ml for both the CPT^ir^ TMH 5006 and CPT^hr^ TMH 5007 strains, showing the absence of cross-resistance between the two antibiotics (Table 1). Of note, although DAP showed *in vitro* activity against CF patient-derived S. aureus strains, the presence of 1% surfactant resulted in an 8- to 32-fold increase in the DAP MIC (up to 8 μg/ml), while the MICs for the other antibiotics, including TLV, remained unchanged (data not shown). These data support previously documented data showing the inactivation of daptomycin in the lung and provide evidence that other anti-MRSA treatment options retain potency.

Of note, we found for strain AMT-0067-21 the presence of internal colonies for which the TLV Etest MIC was 0.19 μg/ml (the MIC was homogeneous up to 0.047 μg/ml) and the broth microdilution MIC was 0.25 μg/ml. Interestingly, the tested MRSA and MSSA strains were not associated with the VISA or DAP-nonsusceptible phenotype. These data altogether provide evidence that TLV retains potency against CF patient-derived S. aureus strains.

### TLV shows bactericidal activity against CF patient-derived MRSA/MSSA isolates.

**FIG 1:**
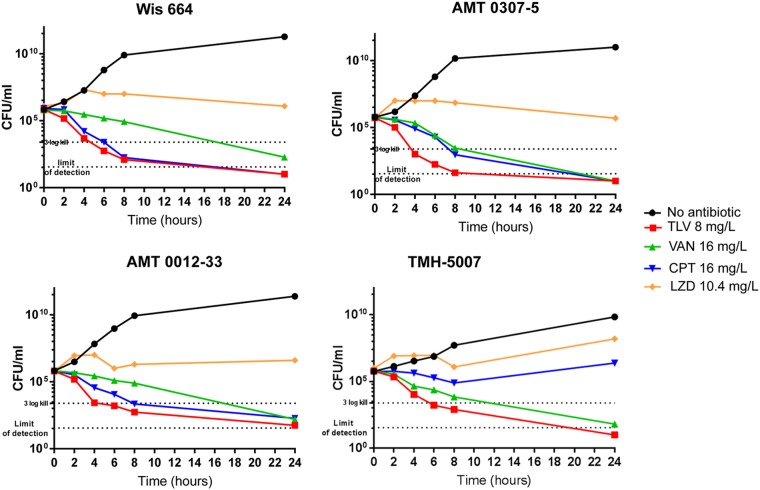
Time-kill curves of CF strains AMT 0307-5 (MSSA), AMT 0012-33, WIS 664 (MRSA), and TMH 5007 (MRSA CPT^r^), determined using the following human peak free serum drug concentrations: 8 mg/liter for TLV, 16 mg/liter for VAN, 10.4 mg/liter for LZD, and 16 mg/liter for CPT. The limit of detection of that assay was 10.

The *in vitro* effectiveness of TLV was also evaluated by time-kill experiments and was compared to that of DAP, VAN, and CPT. The assay was performed in Muller-Hinton broth supplemented with 0.002% polysorbate 80 for TLV and calcium at 50 μg/ml for DAP, and activity against a representative number of CF patient-derived strains, including a strain with CPT^hr^ (TMH 5007), was tested (16–18). TLV showed activity against all the tested strains, including CPT^hr^ strain TMH 5007, in concordance with the TLV MIC values. Moreover, TLV displayed rapid bactericidal activity, with a decrease of more than 3 log_10_ CFU being achieved during the first 4 h of growth (Fig. 1). Similar activity against the CPT^hr^ strain was observed, confirming the absence of cross-resistance or reduced activity. The TLV activity profile at a free serum concentration of 8 mg/liter showed that it performed better than VAN (16 mg/liter), LZD (10.4 mg/liter), and CPT (16 mg/liter) (Fig. 1). Together these data support the potential therapeutic application of TLV for the treatment of S. aureus infections in CF patients.

### *In vitro* selection of TLV-resistant (TLV^r^) mutants.

To determine the fate of mutation selection that can be projected by the potential prolonged use of TLV in CF patients, we investigated the ease of *in vitro* mutation selection in three representative clinical strains from CF patients: the AMT 0114-48, WIS 664, and TMH 5007 (CPT^hr^) strains. S. aureus ATCC 25923 was included as a non-CF patient-derived control strain. These strains were serially passaged in the presence of subinhibitory concentrations of TLV starting at 0.03 μg/ml and escalating up to 3 μg for 40 days. After 15 days, the strains showed an increase in the TLV MIC from 0.064 μg/ml up to 0.25 to 1 μg/ml, followed by a progressive increase up to 3 μg/ml after 40 days of exposure (Table 2). The enhanced MICs of the mutants were stable and unaltered after 10 passages in the absence of TLV. As shown in Table 2, there was a 3- to 4-fold increase in the VAN MIC, which went from 1.5 to 6 μg/ml, and an 8- to 10-fold increase in the DAP MIC, suggesting potential cross-resistance between TLV, VAN, and DAP antibiotics. Moreover, the *in vitro*-derived TLV^r^ mutants grew at lower rate than the parent strains and were defective in growth, requiring 48 h to obtain normal-size colonies on tryptic soy agar (TSA) blood agar plates (data not shown). Important observations were taken from these results: (i) the ease of mutant selection observed in S. aureus ATCC 25913 control strains led us to conclude that TLV mutant resistance is independent of the CF patient background of the strains, and (ii) the likelihood that TLV strains with increased TLV MICs, which was observed for only one strain resistant *in vivo*, AMT-0067-21 (MIC, 0.19 μg/ml), will occur seems rare, considering the fact that this strain represented only 0.3% of the total of 333 strains tested.

**TABLE 2:** MICs of TLV, VAN, and DAP for the parent strains and *in vitro*-derived TLV^r^ mutants obtained by serial passage with subinhibitory concentrations of TLV for 40 days^*a*^

Strain	MIC (μg/ml)					
	Day 0			Day 40		
	TLV	VAN	DAP	TLV	VAN	DAP
ATCC 25913	0.064	1.5	0.25	3	4	8
TMH 5007	0.047	2	0.75	1.5	6	8
AMT 0114-48	0.047	2	0.75	2	6	6
WIS 664	0.032	2	1	1.5	3	6

a As shown, a 3- to 4-fold increase in the VAN MIC (from 1.5 to 6 μg/ml) and an 8- to 10-fold increase in DAP MICs (0.25 to 8 μg/ml) were determined, suggesting potential cross-resistance between TLV, VAN, and DAP antibiotics.

### Genes associated with *in vivo* and *in vitro* TLV resistance.

To investigate the genetic mutations associated with the *in vivo* (MRSA AMT-0067-21) or *in vitro* TLV-resistant mutants, the full genomes of all the strains were sequenced and compared with the sequence of reference strain S. aureus N315 (PATRIC accession number 158879.11). Mutated genes were categorized by function to identify themes of bacterial physiology that may contribute to reduced susceptibility to TLV (Table 3). The most common nonsynonymous single nucleotide polymorphisms (SNP) found by comparing the sequence of each strain with that of its counterpart parental strain were found in cell wall-associated genes, corresponding to *sdrCDE* (encoding a cell wall-associated genes), *tcaA* (encoding a transmembrane protein associated with teicoplanin resistance), and *dltD* (encoding a d-alanine transfer protein). In addition, we found additional nonsynonymous SNPs in various cell wall-associated genes: *spa*, *clfA*, *clfB*, *sdrE*, and *srdD* (Table 3). Moreover, additional SNPs were found in the *in vivo*-derived strain MRSA AMT-0067-21, against which TLV displayed reduced activity, notably, *murE* (a stop codon at position Q), *dltA*, and *vraG* (encoding a bacitracin export permease). Gene deletion occurred for *isdB* (encoding a cell surface receptor for hemoglobin), *mutL* (encoding a DNA mismatch protein), and *fnB* (encoding a fibrinogen binding protein) and in the *yycFG* regulon (encoding a two-component regulatory system) (Table 3). These results may suggest that a decrease in susceptibility to TLV is mainly associated with changes in cell wall-related genes.

**TABLE 3:** Most relevant mutations identified in telavancin-resistant mutants

Gene	Locus	Function^*a*^	SNP					Amino acid change
			TMH 5007	AMT 0114-48	ATCC 25913	WIS 664	Seattle_90	
*acuC*	SA1556	NAD-independent protein deacetylase AcuC	A>T	A>T	A>T	A>T	A>T	I159L
*adh1*	SA0562	Alcohol dehydrogenase	G>T	G>T	G>T	G>T	G>T	K325N
*ald*	SA1531	Alanine dehydrogenase	G>A	G>A	G>A	G>A	G>A	S126L
*capB*	SA0145	Capsular polysaccharide synthesis enzyme Cap5A	T>C	T>C	T>C	T>C	T>C	C81R
*ebhA*	SA1267	Putative staphylococcal surface-anchored protein	A>G	A>G	A>G	A>G	A>G	V4497A
*fni*	SA2136	Isopentenyl diphosphate delta-isomerase, FMN dependent	G>C	G>C	G>C	G>C	G>C	R88G
*gltB*	SA0430	Glutamate synthase (NADPH) large chain	T>C	T>C	T>C	T>C	T>C	V1177A
*holA*	SA1415	DNA polymerase III delta subunit (EC 2.7.7.7)	T>G	T>G	T>G	T>G	T>G	K45T
*hsdM*	SA0391	Type I restriction-modification system, DNA methyltransferase subunit M	T>C	T>C	T>C	T>C	T>C	V387A
*lacE*	SA1992	PTS system, lactose-specific IIB component	T>C	T>C	T>C	T>C	T>C	I365 M
*lytH*, SA1459	SA1458	LytH protein involved in methicillin resistance/*N*-acet	T>C	T>C	T>C	T>C	T>C	I1F
*mutS2*	SA0991	Recombination inhibitory protein MutS2	G>A	G>A	G>A	G>A	G>A	V252I
*pbp2*	SA1283	Multimodular transpeptidase-transglycosylase	G>A	G>A	G>A	G>A	G>A	C197Y
*pbuX*	SA0374	Xanthine permease	T>G	T>G	T>G	T>G	T>G	L231V
*pgm*	SA0730	2,3-Bisphosphoglycerate-independent phosphoglycerate mutase	A>G	A>G	A>G	A>G	A>G	T250A
*rho*	SA1923	Transcription termination factor Rho	T>G	T>G	T>G	T>G	T>G	I48L
*rplA*	SA0496	LSU ribosomal protein L1p (L10Ae)	A>G	A>G	A>G	A>G	A>G	T92A
*rpsQ*	SA2038	SSU ribosomal protein S17p	T>A	T>A	T>A	T>A	T>A	I77L
*sigA*	SA1390	RNA polymerase sigma factor RpoD	C>T	C>T	C>T	C>T	C>T	V253I
*sdrC*	SA0519	Adhesin of unknown specificity SdrC	G>A	G>A	G>A	G>A	G>A	E75K
*sdrD*	SA0520	Adhesin of unknown specificity SdrD	G>T	G>T	G>T	G>T	G>T	D1141Y
*sucA*	SA1245	2-Oxoglutarate dehydrogenase E1 component	T>G	T>G	T>G	T>G	T>G	K842N
*tagG*	SA0594	Teichoic acid translocation permease protein TagG	T>C	T>C	T>C	T>C	T>C	V227A
*tcaA*	SA2146	Membrane protein TcaA, associated with teicoplanin resistance	A>G	A>G	A>G	A>G	A>G	L218P
*thrS*	SA1506	Threonyl-tRNA synthetase	C>T	C>T	C>T	C>T	C>T	G60E
*treP*	SA0432	PTS system, trehalose-specific IIB component	C>G	C>G	C>G	C>G	C>G	A381G
*uvrA*	SA0714	Excinuclease ABC subunit A	T>C	T>C	TA>CG	T>C	TA>CG	L857P
*murQ*	SA0185	*N*-Acetylmuramic acid 6-phosphate etherase					C>T	G257D
*clfA*	SA0742	Clumping factor ClfA, fibrinogen-binding protein					C>A	P208T
*dltA*	SA0793	d-Alanine–poly(phosphoribitol) ligase subunit 1					A>G	K177E
*dltA*	SA0793	d-Alanine–poly(phosphoribitol) ligase subunit 1					G>A	S275N
*dltA*	SA0793	d-Alanine–poly(phosphoribitol) ligase subunit 1					T>C	L318P
*dltA*	SA0793	d-Alanine–poly(phosphoribitol) ligase subunit 1					C>A	D327E
*dltD*	SA0796	Poly(glycerophosphate chain) d-alanine transfer protein DltD					T>A	I264K
*dltD*	SA0796	Poly(glycerophosphate chain) d-alanine transfer protein DltD					T>A	D350E
*spa*	SA0107	Protein A, von Willebrand factor binding protein Spa					CC>TT	G321N
*spa*	SA0107	Protein A, von Willebrand factor binding protein Spa					T>G	K274N
*spa*	SA0107	Protein A, von Willebrand factor binding protein Spa					GT>AG	N234T

a FMN, flavin mononucleotide; PTS, phosphotransferase; *N*-acet, *N*-acetyl; LSU, long subunit; SSU, short subunit.

### *In vitro* TLV mutants are associated with reduced virulence.

In order to determine whether TLV resistance acquired *in vitro* may impact virulence traits in S. aureus, we used Galleria mellonella as an *in vivo* model as it possesses an immune system with reasonable homology to that of vertebrates, and numerous enzymatic cascades akin to complement fixation and blood coagulation occur in the hemolymph, resulting in hemolymph clotting and melanin production as key defense mechanisms against invading microbes. These tissue types are similar to those encountered by S. aureus during invasive infections in humans (19).

**FIG 2:**
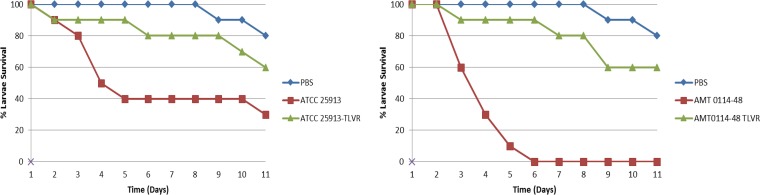
Comparison of virulence of S. aureus strains and their isogenic TLV-resistant mutants in G. mellonella model of infection. The worms were infected at a dose of 10^6^ bacteria per worm. Survival was monitored for 10 days.

Groups of larvae (10/group) were inoculated with a bacterial suspension containing parent strains AMT 0114-48 and WIS 664 and control strain ATCC 25913 and their corresponding TLV-resistant mutants derived *in vitro*, AMT 0114-48 TLV^r^, WIS 664 TLV^r^, and ATCC 25913 TLV^r^ (10^6^ bacteria/worm), as previously described (19). An uninfected control group received phosphate-buffered saline (PBS) treatment to control for multiple injections. Worms were monitored daily, and any deaths that occurred over the next 10 days were recorded. Worms injected with PBS showed 100 to 90% survival at day 8 (Fig. 2), but groups injected with the parent strains (e.g., AMT 0114-48) displayed low survival rates (≤50 to 0%, day 6; Fig. 2). In contrast, groups of worms infected with TLV^r^ strains (e.g., AMT 0114-48 TLV^r^) had a survival rate of 90% at day 6, followed by a survival rate of 60% at day 8. A similar trend was observed for worms injected with the ATCC 25923 strain, although the survival rate was higher (40%) than that for worms injected with CF patient-derived parent S. aureus strains (0 to 20%), while the survival rates for worms injected with the TLV^r^ strains were comparable. These results may suggest that TLV^r^ is associated with changes in virulence fitness.

## DISCUSSION

Infection with S. aureus remains an important concern for CF patients, with a consistently high prevalence occurring in this population. Chronic MRSA infections are associated with worse outcomes, and treatment eradication is a continual clinical challenge. For MRSA pneumonia, the most widely recommended antibiotics are vancomycin and linezolid, with TLV also being approved for this infection type. Although the efficacy of TLV in patients with VAP and HAP has been proven, less is known about its activity and potential efficacy in CF patients with S. aureus pneumonia. In this sense, our study was performed retrospectively to assess the activity of TLV in S. aureus strains isolated from CF patients at three different CF patient centers. The majority of the samples were collected from sputum, and the samples were from both adults and children. The samples were collected during the period from 2015 to 2017. We found that TLV was active against the majority of tested strains, with the exception of one strain against which it showed slightly decreased activity (MICs; 0.19 μg/ml) above the TLV breakpoint.

There are no guidelines or recommendations on the choice of antibiotics for the treatment of pulmonary exacerbations of infections with MRSA in CF patients, resulting in the variable use of active antibiotics between centers. The most frequently used therapies in current practice are trimethoprim-sulfamethoxazole (30%), linezolid (27%), and vancomycin (30%), with LDZ and VAN being the most frequently used among inpatients (15, 27). The pharmacokinetic data available for healthy subjects on TLV intrapulmonary diffusion showed a good penetration of TLV into the epithelial lining fluid and an extensive penetration into alveolar macrophages (20). A clinical trial is currently being conducted (ClinicalTrials.gov registration number NCT03172793 [https://clinicaltrials.gov/ct2/show/NCT03172793]) to evaluate the pharmacokinetic profile of this drug in CF patients, who usually need dose adjustment due to an increase in the volume of distribution and clearance. TLV is not impacted by pulmonary surfactant, unlike daptomycin, making it suitable for use for the treatment of CF-associated lung infections. These observations are consistent with our results. In fact, we demonstrated that TLV has bactericidal activity against the S. aureus strains tested, including those against which CPT and LZD displayed reduced activity (e.g., TMH 5007), which might provide TLV a significant advantage over the drugs currently used to eradicate those strains and prevent future exacerbations.

In the absence of a suitable alternative, TLV has already been used in some CF patients, with successful outcomes (21), supporting its potential role in the management of CF patient-derived MRSA infections. We are unaware of the development of TLV resistance in clinical settings. While direct resistance may be infrequent, modest increases in MICs may be seen in some isolates, as in the isolate described here (TLV MIC, 0.19 μg/ml), and in some strains with VAN and DAP decreased susceptibility. In this context, it was advantageous to gain an understanding of the ease of development of TLV resistance when CF patient-derived strains were extensively exposed to subtherapeutic concentrations of TLV *in vitro*. After 15 days, the strains showed an increase in the TLV MIC from 0.06 μg/ml up to 0.25 to 1 μg/ml, which is above the susceptible breakpoint of 0.12 μg/ml for S. aureus, followed by a progressive increase up to 3 μg/ml after 40 days of exposure. These observations may imply that resistance should be monitored in patients receiving repeated and/or prolonged treatment with TLV. In a previous multistep resistance selection study (22), one stable mutant was obtained from 1 of the 10 MRSA strains after 43 days, correlating with a low mutation frequency. Interestingly, in our study, we were able to demonstrate that TLV resistance selection was independent of the CF patient background of the S. aureus strains, considering the fact that we were able to obtain a TLV^r^ mutant of strain ATCC 25923. We next determined the main genetic changes associated with TLV^r^ by sequencing the full genomes of a representative number of TLV^r^ strains obtained *in vitro* along with the CF patient-derived TLV^r^ strain obtained *in vivo* that showed a modest increase in the TLV MIC (0.19 μg/ml). We found that the TLV^r^ strains harbored common mutations in genes associated with the cell wall (e.g., *murE*, *tagB*) and the cell wall surface (*spa*, *clfB*, *sdrE*). In the TLV^r^ *in vitro* mutants obtained in previous studies by Song et al. (23), most of the differentially expressed genes were also associated with changes in the cell envelope. These findings suggest that although TLV is an agent with a dual mechanism of action (cell membrane, cell wall), the compensatory preferential mutational mechanism seems to be linked to the cell wall to a higher degree. These cell wall mutations may also function in a dual manner to reduce the potency of the mechanism of action of TLV against the cell membrane. This is evidenced by prior studies correlating mutations and reduced VAN susceptibility as a result of VAN treatment (VISA strains) with collaterally reduced DAP activity. Similarly, in our study, the derived TLV^r^ mutants showed cross-resistance, as they showed reduced susceptibility to both VAN (MICs, 3 to 6 μg/ml) and DAP (MICs, 6 to 8 μg/ml).

Nonsynonymous SNPs related to virulence were found in another set of genes (e.g., *sdrE*, *spa*, *clfB*). These changes appeared to suggest that TLV^r^ affects S. aureus virulence fitness, as evidenced in groups of worms infected with TLV^r^ strains (e.g., AMT 0114-48 TLV^r^), which resulted in increased survival rates (90%) compared to those for worms infected with parent strains, which manifested a low survival rate. Previously performed work (22) showed the decrease expression of various virulence factors; however, their functional role was not demonstrated.

In conclusion, the present data suggest that TLV is active against CF patient-derived MRSA strains independently of associated CPT resistance mechanisms and may constitute a new option for the treatment of MRSA infections in CF patients.

## MATERIALS AND METHODS

### Clinical CF strains.

The clinical strains used in this study were isolated from cultures of CF patient sputum. A collection of strains comprising either the wild-type or the small-colony-variant (SCV) phenotype was obtained from three academic medical institutions with large CF populations: the Center of Global Infectious Diseases (Seattle, WA), UW Health (Madison, WI), and the Houston Methodist Hospital (Houston, TX).

### Susceptibility testing.

Susceptibilities to telavancin (TLV), ceftaroline (CPT), daptomycin (DAP), linezolid (LZD), and vancomycin (VAN) were determined by Etest (bioMérieux). TLV MICs were also determined by the microdilution method in cation-adjusted Mueller-Hinton broth II (MHBII) supplemented with polysorbate 80 (0.002%) following CLSI guidelines (24).

### Time-kill analyses.

Time-kill analyses were performed on 4 representative CF patient-derived strains following CLSI guidelines using human free drug maximal concentrations for TLV (8 mg/liter for a 750-mg dose), VAN (16 mg/liter for a 1,000-mg dose), LZD (10.4 mg/liter for a 600-mg dose), and CPT (16 mg/liter for a 600-mg dose) in 24-well microplates (16–18, 25). The numbers of CFU were counted by plating a sample on tryptic soy agar (TSA) at 0, 2, 4, 6, 8, and 24 h. Results were expressed as the number of CFU per milliliter versus time.

### TLV *in vitro* mutant selection.

*In vitro* mutant selection was attempted with a representative number of CF patient-derived strains. S. aureus ATCC 25923 was included as a non-CF patient-derived reference strain. Strains and controls were exposed to subinhibitory concentrations of TLV in Mueller-Hinton broth for 40 days by progressive passages to recover nonsusceptible TLV strains.

### Assessment of virulence between TLV-susceptible and -resistant mutants in a wax worm model of infection.

Groups of Galleria mellonella larvae (10/group) were inoculated with 10 μl of a bacterial suspension of strains AMT 0114-48, AMT 0114-48 TLV^r^, ATCC 25923, and ATCC 25923 TLV^r^ containing 1.5 × 10^6^ CFU/ml, as previously described (26). The inoculum was administered directly to the larval hemocoel through the last left proleg as previously described (19, 25). Every trial included a group of 10 untreated larvae as an uninfected control group and 10 larvae injected with PBS as a method control. The experiments were performed in at least three independent trials. Injected insects were held at 37°C and monitored over 7 days. By day 7, pupa formation in the surviving larvae was recorded.

### Genomic characterization and whole-genome sequencing.

DNA from the different strains was prepared using a DNeasy blood and tissue kit (Qiagen). Libraries were prepared from purified DNA using a Nextera XT DNA library preparation kit (Illumina) and sequenced with HiSeq 2000 instruments at the Epigenetics and Genomic Laboratory at Weill Cornell University, New York, NY. Genomes were assembled, and SNPs were identified by comparison to the sequence of S. aureus N315 (GenBank accession number BA000018) using the Lasergene (version 14) suite. The reads were aligned against the sequence of N315 (PATRIC accession number 158879.11) and analyzed using PATRIC variation service 24, which uses the BWA-mem algorithm as the read aligner (https://arxiv.org/abs/1303.3997) and FreeBayes as the SNP caller (https://arxiv.org/abs/1207.3907).
